# Mathematical models of the solid cancer induced by atomic bomb and the spontaneous cancer in the daily life—Proposal of a new medical treatment for cancers

**DOI:** 10.1002/cnr2.1697

**Published:** 2022-08-12

**Authors:** Hiroshi Baba, Akihiko Yokoyama

**Affiliations:** ^1^ Osaka University Osaka Japan; ^2^ Institute of Science and Engineering Kanazawa University Kanazawa Japan

**Keywords:** atomic bomb, mathematical model, radiation‐induced cancer, solid cancer, spontaneous cancer

## Abstract

**Background:**

A mathematical model of the radiation−induced cancer was devised to explain the change of incidence rates pursued by Radiation Effect Research Foundation for 25 years.

**Aim:**

The aim of this work is construction of mechanisms of radiation‐induced cancer and cancers observed in the daily life.

**Methods and results:**

First, we found a way to separate spontaneous cancers from radiation‐induced cancers observed among atomic‐bomb victims in Hiroshima and Nagasaki districts by using a constructed algorithm. The isolated incidence rates of radiation‐induced cancers were reproduced by a two‐stage model mechanical collision of impinging radiation with cells and succeeding mutation of the damaged cell to cancer. This model satisfactorily reproduced observed solid cancer incidence rates. We further attempted to construct a mathematical model for the age‐dependence of spontaneous cancers appearing in the daily life and concluded that the cancer should be generated at cell division.

**Conclusion:**

With these findings, we reached to a conclusion that cancers may be suppressed by eliminating damaged cells with mild–dose radiation.

## 1 INTRODUCTION

Fukushima–Daiichi nuclear power plant accident in 2011 evoked a great interest on the exposure, particularly internal exposure among the people living in Japan at the moment. However, the object of discussion was concentrated on the allowable exposure level and any quantitative discussion on the radiation injury was never attempted.

The typical injury caused by radiation is the cancer, particularly that observed among Hiroshima−Nagasaki atomic−bomb survivors. Solid cancers except for cancer of blood, malignant tumor etc. were energetically followed up by a group^1^ mainly consisting of the members of Radiation Effects Research Foundation (RERF) from 1975 to 1999. This valuable report is the only one quantitative document on the incidence rates for solid cancers among Hiroshima−Nagasaki atomic bomb survivors.

Several investigations^2^ are carried out using the RERF data. However, their interests were concentrated on the comparison of radiation risks among cohorts with respect to sex, age at the time of the bombing, radiation dose or dose rate, and so forth. In such a study, it is conceivable that the fundamental part of the mechanism of carcinogenesis has been washed out. None of the proposed models succeeded in giving quantitative explanation for the RERF data.^1^ Therefore, we may have to look for a different approach to the carcinogenesis.

In 1957, Armitage and Doll published a multistage model (AD model)^3^ for carcinogenesis of radiation‐induced cancers. It consists of damaged intermediate cells formed by irradiation of healthy cells and malignant cells produced by transformation of the unstable intermediate cells repeated for several times until the malignant cell reaches to the cancer stage. The multistage was necessary to explain a long lag time before cancer becomes tangible.

Though the AD model is quite a reasonable model to explain radiation‐induced cancer, it has some weak points; for instance, we cannot tell how many transformations are required to reach cancer and we are not able to detect malignant cells at any intermediate stage. Introduction of such unknown stages results in bringing many free parameters into the model, which makes interpretation of observed data somewhat ambiguous.

This shortcoming can be removed by omitting the undetectable multistage transformations after the irradiation process in the multistage model. The long lag after formation of intermediate cells are explained as the time required for statistical mutation of the damaged cell to cancer cell. Thus, multistage AD model were settled as the most simple two‐stage model.^4^, ^5^, ^6^, ^7^, ^8^, ^9^, ^10^, ^11^

We noticed the analogies between creation of intermediate cells by radiation of A‐bombing and the production of radioactive nuclides by an accelerator, and between mutation of generated intermediate cells and disintegration of radioactive nuclides.

Then, we attempted reproducing the incidence rates reported by RERF gropu.^1^ Solid cancers caused by radiation are clinically indistinguishable from cancers naturally occurring in the daily life. The incidences given in reference 1 contain those of the spontaneous cancers. Therefore, we first built a mathematical model for reported incidence rates containing the spontaneous cancer and devised a method deducing the magnitude of the spontaneous cancer by means of thus derived formula for the model.

Thus, we could estimate the net incidence rate for the radiation‐induced cancer. Then, the above radiochemical cancer model was applied to the net incidence data.

Finally, we attended to the age‐dependence of spontaneous cancer incidence rate to make a model of spontaneous cancer model. We noticed the nearly exponential distribution of the death rate by cancer versus age and concluded that the mutation takes place at the moment of cell division. With thus constructed whole story of cancers, we found the possibility of a harmless medical treatment of cancers using low‐level radiation.

## 2 RISK OF SOLID CANCERS

Sudden mutation was shown by Manabe et al. to explain with a simple reaction rate formula^12^, ^13^ which does not require consideration of existence of the latent period before mutation takes place. On the contrary, solid cancers induced by radiation requires quite long latent periods. To reproduce such latent periods, the model needs to be divided into two steps, the process giving damage to cells by radiation exposure and the transformation process of damaged cells to cancer cells.

Mechanism of the two‐stage radiation‐induced cancer^4^, ^5^, ^6^, ^7^, ^8^, ^9^, ^10^, ^11^ consists of two fundamental radiochemical processes, mechanical collision between an impinging neutron, electron or photon and electrons bound to a chemical bond to produce unstable fragments, and a statistical event equivalent to the decay of radioactive nuclides.

This principle was applied to the research data^1^ on the incidence of solid cancers among Hiroshima−Nagasaki survivors to construct a mathematical model. The first step producing radiation damage is a simple physical process and can be treated essentially likewise as the reaction rate formula of sudden mutation1.^12^, ^13^ The second step for the latent period is a statistical process following the first‐order reaction, and can be treated as the radioactive decay, that is, the growth rate equation for the daughter nuclide in the radioactive decay.

Normal cells received radiation exposure are divided into the following four groups.

- cells receiving no changes
- cells receiving damage leading to cancers
- cells receiving damage not leading to cancers
- cells proceeding to death.

The cells of group C can be recognized phenomenally as the same member of group A. The member of the reaction channel D should be negligibly small compared to members of other groups which contain various channels so that one should consider normal cells *N*_n_ and damaged cells *N*_d_ leading to cancer in the equation.

The number of normal cells remaining just after bombing is given by (1 − *γD*)*N*_0_ with original number of normal cell *N*_0_ and the radiation dose at bombing *D*, where *γ* is the probability of producing damage. The number of damaged cells is given by *γD N*_0,_

Then the change of damaged cells is written as

(1) $$dN_{d}/dt=\alpha \left\{\left(1-\mathrm{\gamma D}\right)N_{0}-N_{d}\right\}-\beta N_{d},$$

Here, α and β are reaction rate constants while γ *D* is the rate of damaged cells produced at the moment of bombing to the total cells. The first term of the right side of Equation (1) is the number of normal cells at time *t* after bombing. The second term represents the relaxing process of damaged condition by radiation and *β* gives the rate constant of relaxation process.

Equation (1) is transformed for one normal cell as follows

(2) $$\delta Ν\left(t\right)/\delta t=\alpha \left(1-\gamma \Delta \right)-\left(\alpha +\beta \right)N,$$

where *N* = *N*_d_/*N*_0_. The solution of Equation (2) is

(3) $$N=f\left(1-e^{-\mu \cdot t}\right)+\mathrm{\gamma D}\;e^{-\mu \cdot t},$$

where μ=α+β and $f=\left(\alpha /\mu \right)\left(1-\gamma \Delta \right)$.

The continuous time variable is now replaced with an integer *n* corresponding to 1 year. That is, 1 year is regarded to be the unit of time. The factor for the latent period is introduced into the equation

(4) $$N_{c}\left(n\right)=\sum_{i=1}^{n-1}N\left(i\right)\left[1-e^{-\Lambda \left(n-i\right)}\right],$$

where *N*_c_(*n*) is the number of cancer cells at the *n*th year after bombing and Λ is the rate constant of mutation.

The incidence rate is given as the number of patients per 10^5^ populations, so that we must replace the concept of the number of damaged cell by the number of patients. This procedure may be allowed because the number of persons received multiple damages is expected to be negligibly small considering a quite low geometrical cross section for contact of radiation.

We can set a condition Λ<μ from the characteristics requested to Λ and Equation (4) is transformed as follows;

(5) $$N_{C}\left(n\right)=f\left[n-1-\frac{1-e^{-\Lambda \cdot n}}{1-e^{-\Lambda }}+B\left\{\frac{1-e^{-\mu \cdot n}}{1-e^{-\mu }}-\frac{e^{-\Lambda \cdot n}-e^{-\mu \cdot n}}{1-e^{-\left(\mu -\Lambda \right)}}\right\}\right]$$

Here, *B* is a secondary parameter like μ and *f* defined by

(6) $$B=\left(\mathrm{\gamma D}-f\right)/f.$$

As is obviously seen, the incidence rate *N*_c_ is expressed with four parameters *f*, μ, Λ, and *B*. However, *f* is a scaling factor and not an independent parameter excluded in the fitting procedure. The two parameters *B* and γD obey the restrictions that *B* > −1 and γD < 1, respectively.

The incidence rates given in the RERF report^1^ are the sum of people affected by cancers for 5 years. Therefore, Equation (5) is written as

(7) $$Q\left(n\right)=\sum_{j=n-2}^{n+2}N_{C}\left(j\right)$$

to compare the model with observed data.

## 3 INCIDENCE OF STOMACH CANCER

Among 33 cancers given in reference 1, the incidence rate of cancers of stomach (for Male and Female), cervix uteri and gallbladder (for Female) are decreasing after 30 years since bombing. This trend was never reproduced by changing values of above three free parameters. This implies that we need to introduce another function guaranteeing decrease of the incidence with time.

In 2001, a report was published that no stomach cancer patients appeared among people not infected with pylori bacteria,^14^, ^15^ while occurrence of stomach cancer was observed among people infected with the bacteria.^16^ Suppression of cancers may be attributed to the immunity function of removing bacteria. Decrease of incidence is also observed among gallbladder, cervix uteri, and so forth.^1^ Therefore, we must find a universal type of immunity function for incidence.

Recently, Sato et al. of National Institute of Health, USA reported that they succeeded^17^ in reducing tumor of cancers of lung and large intestine by depleting regulatory T cells to activate the action of immune cells with generated heat by irradiation of near−infrared rays. This would support an idea of coexistence of immune and anti−immune cells, and anti−immune cells are rather weak against outer actions including radiation.

Destruction of anti−immune cells by radiation is the first−order reaction and controlled by the same equation for the decay of radioactive nuclides. Then, the incidence risk of stomach cancer at *n* years after bombing is given by

(8) $$N_{s}\left(n\right)=N_{c}\left(n\right)e^{-\mathrm{\varepsilon n}}.$$

Then, the incidences for 5 years is given by

(9) $$Q\left(n\right)=\sum_{j=n-2}^{n+2}N_{C}\left(j\right)e^{-\varepsilon \cdot j}$$

We first surveyed the structure of the $\chi^{2}$ values in the phase space constructed with three parameters, μ, Λ, and *B*, before starting the search of the best−fit condition, where

(10) $$\chi^{2}=\sum_{j=1}^{5}{\left\{\frac{Q_{\mathrm{cal}}\left(5j+28\right)-Q_{\mathrm{obs}}\left(5j+28\right)}{Q_{\mathrm{obs}}\left(5j+28\right)}\right\}}^{2}$$

**FIGURE 1:**
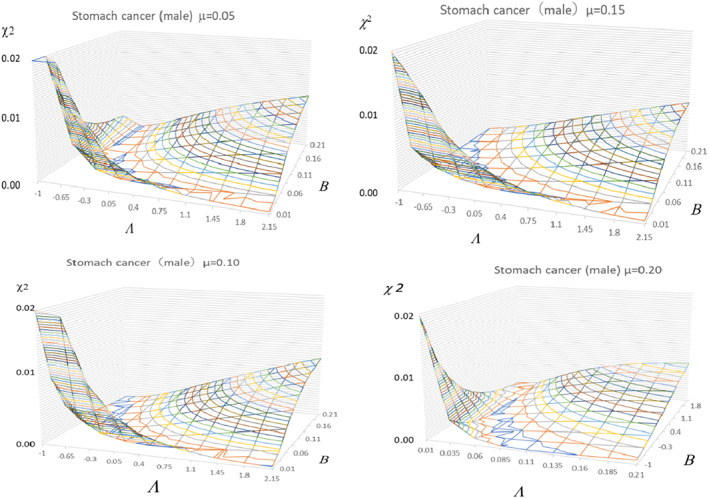
Contour maps of the two‐dimensional structure of the χ^2^ values versus Λ and *B*

$\chi^{2}$ is a measure of deviation of the calculated value from the observed one. The vest fit is obtained by a minimum spot of $\chi^{2}$ in the phase space of the above three parameters. The minimum spot is searched by the least squares method with a computer. However, obtained two−dimensional contour map of $\chi^{2}$ revealed very shallow valley without any clear minimum spot as shown in Figure 1 for stomach cancer. Therefore, the least square fitting procedure using contour map of $\chi^{2}$ does not work for the present case because the final answer primarily depends on initial input data and does not necessarily merge to the true destination.

However, we can introduce another severe condition that μ and Λ must stay, respectively, at a common value, respectively, throughout reported 33 cancers, because the processes described by Equations (1), (2), and (8) are purely physical events and so they should not be affected by the difference of internal organs. Then we continued a steady and persistent calculation of *Q*_cal_ by changing the values of three parameters little by little under the above−mentioned condition until somewhat satisfied fitness was obtained as shown in Tables 1 and 2, together with deduced values of the parameters in Table 3.

**TABLE 1:** Observed and calculated incidence rate (number of incidence per 10^5^ population) during 5 years after the bombing for Hiroshima–Nagasaki atomic bomb survivors (Male)

Solid cancer		Elapsed time after the bombing (*y*)					Spontaneous cancers^10^
		31–35	36–40	41–45	46–50	51–55	
All solid cancers	obs	193.1	214.9	243.0	249.8	253.6	136.7
	cal	196.0	219.2	236.4	248.0	254.8	
Oral cavity	obs	3.3	4.2	4.8	5.1	5.8	1.6
	cal	3.4	4.1	4.7	5.3	5.8	
Esophagus	obs	8.2	8.2	9.3	9.6	10.6	7.2
	cal	7.6	8.6	9.4	9.9	10.3	
Stomach	obs	80.5	77.2	79.1	70.9	64.1	56.5
	cal	80.3	79.7	76.2	70.9	64.7	
Colon	obs	9.3	13.4	18.9	28.3	30.4	4.4
	cal	9.2	13.9	19.4	25.5	32.3	
Rectum	obs	9.1	11.9	14.1	16.4	18.4	5.6
	cal	9.3	11.6	13.9	16.3	18.7	
Liver	obs	14.1	19.6	25.0	26.0	24.3	11.9
	cal	13.6	21.0	24.6	25.4	24.4	
Gallbladder	obs	4.4	5.6	6.8	6.7	6.6	3.2
	cal	4.4	5.6	6.4	6.8	6.8	
Pancreas	obs	6.8	7.7	9.1	8.9	8.9	5.6
	cal	6.7	7.8	8.6	9.0	9.3	
Lung	obs	25.0	30.5	35.7	36.5	37.4	18.0
	cal	25.7	29.9	33.6	36.7	39.2	
Prostate	obs	5.2	6.5	7.9	9.2	12.1	2.1
	cal	5.3	6.7	8.1	9.6	11.1	
Kidney	obs	2.7	3.4	4.9	5.6	6.3	1.0
	cal	2.8	3.7	4.6	5.5	6.4	
Bladder	obs	5.7	7.8	7.6	8.0	8.7	2.2
	cal	6.0	6.9	7.7	8.3	8.8	
Brain	obs	20	2.6	2.9	2.4	2.4	—
	cal	2.2	2.4	2.5	2.6	2.6	
Thyroid	obs	0.7	1.3	1.6	1.3	1.5	—
	cal	0.8	1.0	1.3	1.5	1.7	

**TABLE 2:** Observed and calculated incidence rate (number of incidence per 10^5^ population) during 5 years after the bombing for Hiroshima–Nagasaki atomic bomb survivors (Female)

Solid cancer		Elapsed time after the bombing (*y*)					Spontaneous cancers^10^
		31–35	36–40	41–45	46–50	51–55	
All solid cancers	obs	134.0	141.5	152.3	152.8	155.1	103.6
	cal	133.0	144.4	151.1	153.8	153.4	
Oral cavity	obs	1.3	1.9	2.0	1.8	2.0	1.5
	cal	1.3	1.6	1.8	2.0	2.3	
Esophagus	obs	1.8	1.7	1.5	1.4	1.4	2.1
	cal	1.8	1.7	1.6	1.4	1.3	
Stomach	obs	38.6	35.9	35.0	29.3	25.3	35.3
	cal	39.2	36.8	33.3	29.4	25.3	
Colon	obs	7.7	10.1	13.1	16.9	17.7	4.8
	cal	8.0	10.5	13.1	15.7	18.2	
Rectum	obs	6.2	6.8	7.7	8.5	8.7	4.7
	cal	6.1	7.0	7.7	8.3	8.8	
Liver	obs	5.3	5.7	6.7	7.2	7.4	6.7
	cal	5.4	6.1	6.6	7.0	7.3	
Gallbladder	obs	4.7	6.0	6.7	6.0	5.3	4.2
	cal	4.7	6.0	6.4	6.1	5.5	
Pancreas	obs	3.8	4.5	5.2	5.0	5.1	3.0
	cal	3.8	4.5	4.9	5.1	5.3	
Lung	obs	7.5	9.2	10.3	10.1	11.4	6.6
	cal	7.7	8.9	9.9	10.7	11.3	
Breast	obs	17.5	21.4	26.6	28.8	32.8	5.4
	cal	17.9	21.8	25.5	29.1	32.6	
Cervix uteri	obs	13.8	12.5	10.1	7.8	7.6	—
	cal	13.6	12.1	10.4	8.7	7.1	
Corpus uteri	obs	1.6	2.9	3.2	3.9	4.6	11.0
	cal	1.8	2.4	3.2	4.0	4.9	
Ovary	obs	1.8	4.3	5.7	5.9	6.6	2.5
	cal	3.8	4.6	5.3	6.0	6.6	
Kidney	obs	1.0	1.5	1.7	2.0	2.3	0.5
	cal	0.9	1.3	1.7	2.l	2.6	
Bladder	obs	1.5	1.8	1.8	2.0	2.0	1.1
	cal	1.6	1.8	1.9	1.9	1.9	
Brain	obs	1.4	1.8	2.2	1.8	1.9	—
	cal	1.6	1.8	1.9	1.9	1.9	
Thyroid	obs	2.4	3.4	5.2	6.4	5.9	—
	cal	2.5	3.5	4.6	5.7	7.0	

**TABLE 3:** Parameters for solid cancers including spontaneous cancers

Solid cancer		μ,Λ,β	*B*	ε	*f* (×10^−5^)	*γD* (×10^−5^)	*α* (×10^−5^)	Latent period (*y*)
All solid cancers	M	$\left(\begin{array}{c} \mu =0.1\\ \Lambda =0.05\\ \beta ≓\mu \end{array}\right)$	0.1	0.024	5.37	5.91	0.54	6
	F			0.03	4.45	4.89	0.45	
Oral cavity	M			0.01	0.06	0.06	0.006	
	F			0.01	0.02	0.02	0.002	
Esophagus	M			0.022	0.20	0.22	0.020	
	F			0.054	0.13	0.14	0.013	
Stomach	M			0.048	4.86	5.35	0.49	
	F			0.059	3.41	3.76	0.34	
Rectum	M			0.002	0.12	0.14	0.012	
	F			0.019	0.14	0.16	0.014	
Liver	F			0.022	0.14	0.15	0.014	
Lung	M			0.016	0.54	0.60	0.054	
	F			0.018	0.17	0.19	0.017	
Prostate	M			0	0.07	0.07	0.007	
Bladder	M			0.018	0.14	0.15	0.014	
	F			0.03	0.06	0.06	0.006	
Brain	M			0.03	0.07	0.08	0.007	
	F			0.03	0.06	0.06	0.006	
Thyroid	M			0	0.01	0.01	0.001	
Cervix uteri	F			0.07	1.70	1.87	0.17	
Ovary	F			0.01	0.07	0.07	0.007	
Breast	F			0.007	0.28	0.31	0.028	
Kidney	M	$\left(\begin{array}{c} \mu =0.05\\ \Lambda =0.01\\ \beta ≓\mu \end{array}\right)$	0.1	0.01	0.19	0.21	0.01	15
	F			0	0.05	0.05	0.003	
Thyroid	F			0	0.12	0.14	0.006	
Colon	F			0.01	0.55	0.60	0.028	
Corpus uteri	F			0	0.09	0.10	0.005	
Pancreas	M			0.035	1.05	1.16	0.053	
	F			0.035	0.60	0.66	0.030	
Colon	M		−0.5	0	0.84	0.42	0.042	18
Gallbladder	M		−0.7	0.05	2.96	0.89	0.15	22
	F		−0.9	0.08	4.15	1.41	0.21	25
Kidney	M		−0.98	0.07	40.57	0.81	2.03	27

Table 3 indicates the 33 cancers are divided into two groups. Majority of them satisfy our speculation, while the remain minor group gathered with a set of slightly smaller common values of the three parameters, μ, Λ, and *B*. The reason why they are separated to two groups are not clear.

## 4 ESTIMATION OF SPONTANEOUS INCIDENCES

Observed incidence rates in Tables 1 and 2 include spontaneous incident rates induced with no radiation effect as stated before. Unfortunately, we are unable to clinically distinguish the radiation−induced and spontaneous cancers. Mixing of the spontaneous incidence might give us a false view of the mechanism of cancer.

There are two difficulties for searching spontaneous incidence rates among published data. The first problem is that it is practically impossible to know the exact incidence time for spontaneous cancers among general people contrary to carefully traced inhabitants in Hiroshima−Nagasaki area. The second difficulty is the difference in the age composition of population. The peak of age composition of population in Hiroshima and Nagasaki is considered to shift toward younger age compared to the national population because aged people passed away to a greater extent due to their physical weakness under such a severe circumstance. The death rates in reference 18 stays at a very low level under 50 years old but rapidly increases with age thereafter. This would result in smaller incidence rates (death rates) under the influence of radiation. Spontaneous incidence rates under the normal condition were estimated using total number of incidence by the spontaneous cancer^18^ and the national population^19^ ranging from 1972 to 1975 as shown in the last column of Tables 1 and 2.

The method to subtract the spontaneous incidence rates from the radiation−induced data must be found as a statistical way if possible. First, let us transform Equation (9) by introducing a minor approximation of changing the exponential factor $e^{-\varepsilon \cdot j}$ to $e^{-\varepsilon \cdot n}$ and taking the exponential factor out of the summation with respect to *j*. We further introduce a free parameter *Q*_sc_ as the spontaneous incidence rate and a physical quantity ξ(*n*) defined by
(11)

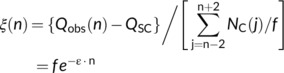

These procedure leads to an equation;

(12) $$\ln\;\xi \left(n\right)=\ln\;f-\varepsilon \cdot n.$$

**FIGURE 2:**
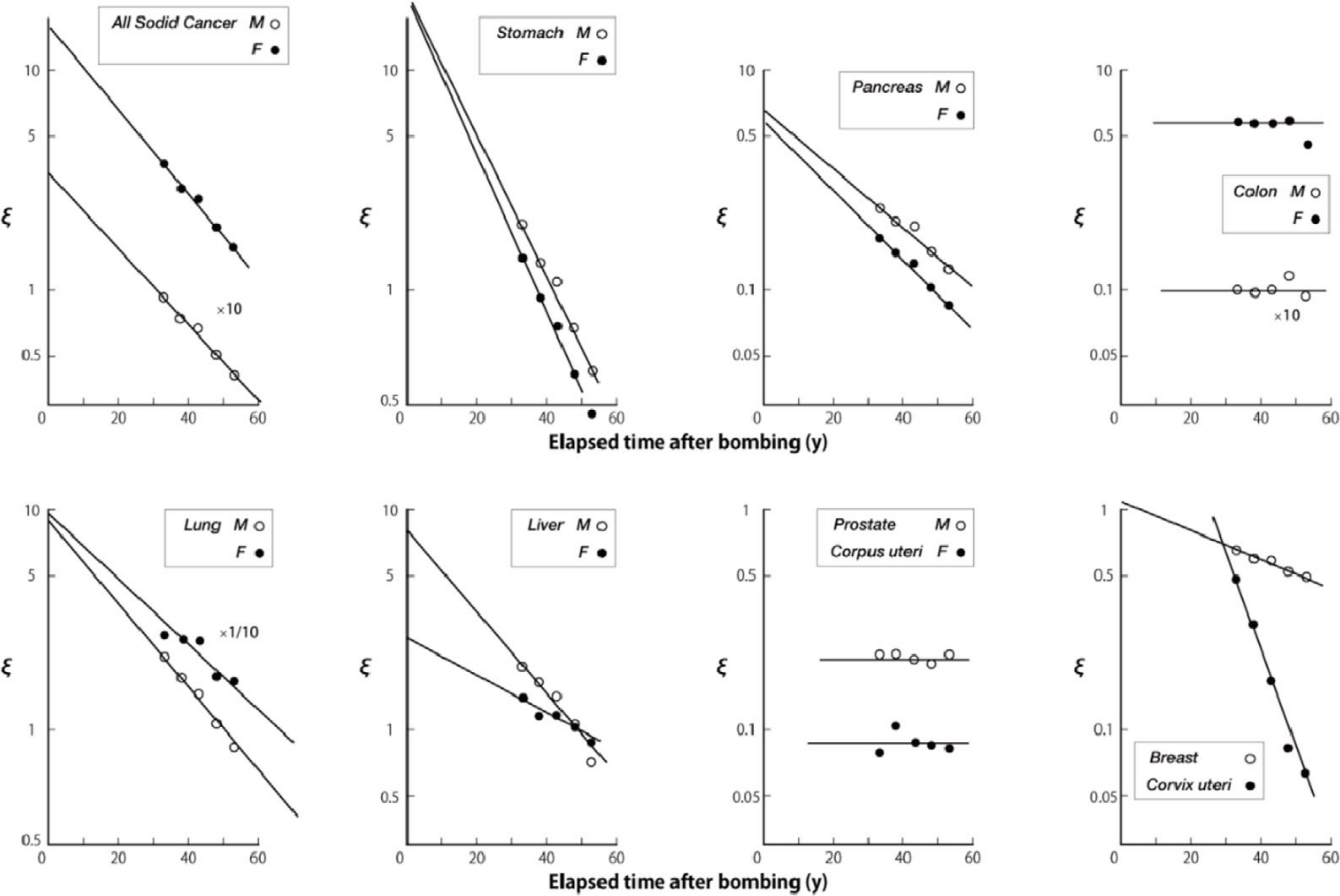
Linear relation giving the contents of a spontaneous cancer Equation (11)

Equation (12) now requires that ln ξ (*n*) should show a linear dependence with respect to time. That is, the spontaneous incidence rate *Q*_sc_ is determined so as to satisfying Equation (12) as shown in Figure 2. Linear relationship was obtained for all cancers and then *Q*_sc_ was deduced. The results are shown in Figure 1 for half of the reported 33 cancers and the deduced *Q*_sc_ values of all cancers are given in the last column of Tables 4 and 5. The net incidence rate of radiation−induced cancer was obtained by subtracting thus deduced *Q*_sc_ values from the observed incidence rate.

**TABLE 4:** Observed and calculated incidence rate (number of incidence per 10^5^ populations) of net radiation‐induced solid cancers (first group)

Solid cancer			Elapsed time after the bombing (*y*)					$Q_{\mathrm{SC}\left(\times 10^{‐5}\right)}$
			31–35	36–40	41–45	46–50	51–55	
Oral cavity	M	obs	2.3	3.2	3.8	4.1	4.8	1.0
		cal	2.5	3.1	3.7	4.2	4.7	
	F	obs	0.8	1.4	1.5	1.3	1.5	0.5
		cal	1.2	1.4	1.4	1.5	1.5	
Esophagus	M	obs	4.2	4.2	5.3	5.6	6.6	6.0
		cal	3.6	4.5	5.3	6.1	6.8	
	F	obs	1.0	0.9	0.7	0.6	0.6	0.8
		cal	1.0	0.9	0.7	0.6	0.5	
Stomach	M	obs	40.5	37.2	39.1	30.9	24.1	40.0
		cal	40.6	38.9	35.2	30.9	26.0	
	F	obs	28.6	25.9	25.0	19.3	15.3	10.0
		cal	28.6	26.0	22.3	18.9	15.4	
Lung	F	obs	5.5	7.2	8.3	8.1	9.4	2.0
		cal	5.3	6.6	7.9	9.2	10.5	
Pancreas	M	obs	4.8	5.7	7.1	6.9	6.9	2.0
		cal	4.7	5.6	6.2	6.6	6.9	
	F	obs	3.5	4.2	4.9	4.7	4.8	0.3
		cal	3.6	4.1	4.5	4.7	4.8	
Kidney	M	obs	1.9	2.6	4.1	4.8	5.5	0.8
		cal	2.0	2.8	3.7	4.6	5.6	
	F	obs	1.0	1.5	1.7	2.0	2.3	0
		cal	0.9	1.3	1.7	2.0	2.6	
Breast	F	obs	13.5	16.9	21.5	24.9	27.8	4.0
		cal		17.3	21.0	24.4	27.6	
Cervix uteri	F	obs	9.8	8.5	6.1	3.8	3.6	4.0
		cal	9.3	7.8	6.2	4.7	3.5	
Bladder	M	obs	3.5	5.6	5.4	5.8	6.5	1.1
		cal	3.6	4.4	5.2	5.9	6.5	
	F	obs	1.0	1.3	1.3	1.5	1.5	0.5
		cal	1.0	1.2	1.3	1.4	1.4	
Thyroid	M	obs	0.4	0.9	1.3	1.0	1.2	0.3
		cal	0.4	0.6	0.8	1.0	1.2	
Brain	M	obs	1.5	2.1	2.4	1.9	1.9	0.5
		cal	1.7	2.0	2.0	2.0	2.0	
	F	obs	0.9	1.3	1.7	1.3	1.4	0.5
		cal	1.0	1.2	1.4	1.5	1.5	
Liver	F	obs	2.8	3.2	4.2	4.7	4.9	2.5
		cal	2.7	3.4	4.1	4.7	5.0	
Corpus uteri	F	obs	1.6	2.9	3.2	3.9	4.6	0
		cal	1.8	2.4	3.2	4.0	4.9	

**TABLE 5:** Observed and calculated incidence rate (number of incidence per 10^5^ populations) of net radiation‐induced solid cancers (second group)

Solid cancer			Elapsed time after the bombing (*y*)					*Q*_SC_(×10^−5^)
			31–35	36–40	41–45	46–50	51–55	
Rectum	F	obs	1.5	2.1	3.0	3.8	4.0	4.7
		cal	1.4	2.1	2.8	3.6	4.5	
	M	obs	3.5	6.3	8.5	10.8	12.8	5.6
		cal	3.8	5.7	8.1	10.7	13.5	
Prostate	M	obs	3.1	4.4	5.8	7.1	10.0	2.1
		cal	3.0	4.3	5.8	7.5	9.4	
Ovary	F	obs	1.2	1.7	3.1	3.3	4.0	2.6
		cal	1.2	1.8	2.5	3.4	4.3	
All solid cancers	M	obs	73.1	94.9	123.0	129.8	133.6	120.0
		cal	73.0	97.8	116.5	129.6	137.4	
	F	obs	30.0	37.5	48.3	48.8	51.1	104.0
		cal	29.9	39.1	45.5	49.3	50.9	
Lung	M	obs	10.0	15.5	20.7	21.5	22.4	15.0
		cal	10.1	15.3	19.3	21.9	23.4	
Liver	M	obs	9.1	14.6	20.0	21.0	19.3	5.0
		cal	9.2	14.3	18.1	20.7	22.4	
Colon	M	obs	4.7	8.8	14.3	23.7	25.8	4.6
		cal	4.7	8.9	14.0	20.0	26.8	
	F	obs	2.8	5.2	8.2	12.0	12.8	4.9
		cal	2.7	5.2	8.2	11.7	15.7	
Gallbladder	M	obs	3.9	5.1	6.3	6.2	6.1	0.5
		cal	3.9	5.3	6.1	6.2	6.0	
	F	obs	4.2	5.5	6.2	5.5	4.8	0.5
		cal	4.1	5.4	5.8	5.6	5.2	
Thyroid	F	obs	1.9	2.9	4.7	5.9	5.4	0.5
		cal	2.0	3.5	4.2	5.1	5.7	

Now, we return to Equation (9) and try to find the best choice of free parameters so as to reproduce the net incidence rate, {*Q*_obs_(*n*) – *Q*_sc_} which are given in Tables 4 and 5, together with deduced spontaneous incidence rates *Q*_sc_. Temporarily obtained values of parameters for the whole incidence rate in Tables 4 and 5 were considered as the initial guess in the fitting procedure for the incidence rates of net radiation−induced cancers given at the upper line for each cancer in Tables 4 and 5.

**FIGURE 3:**
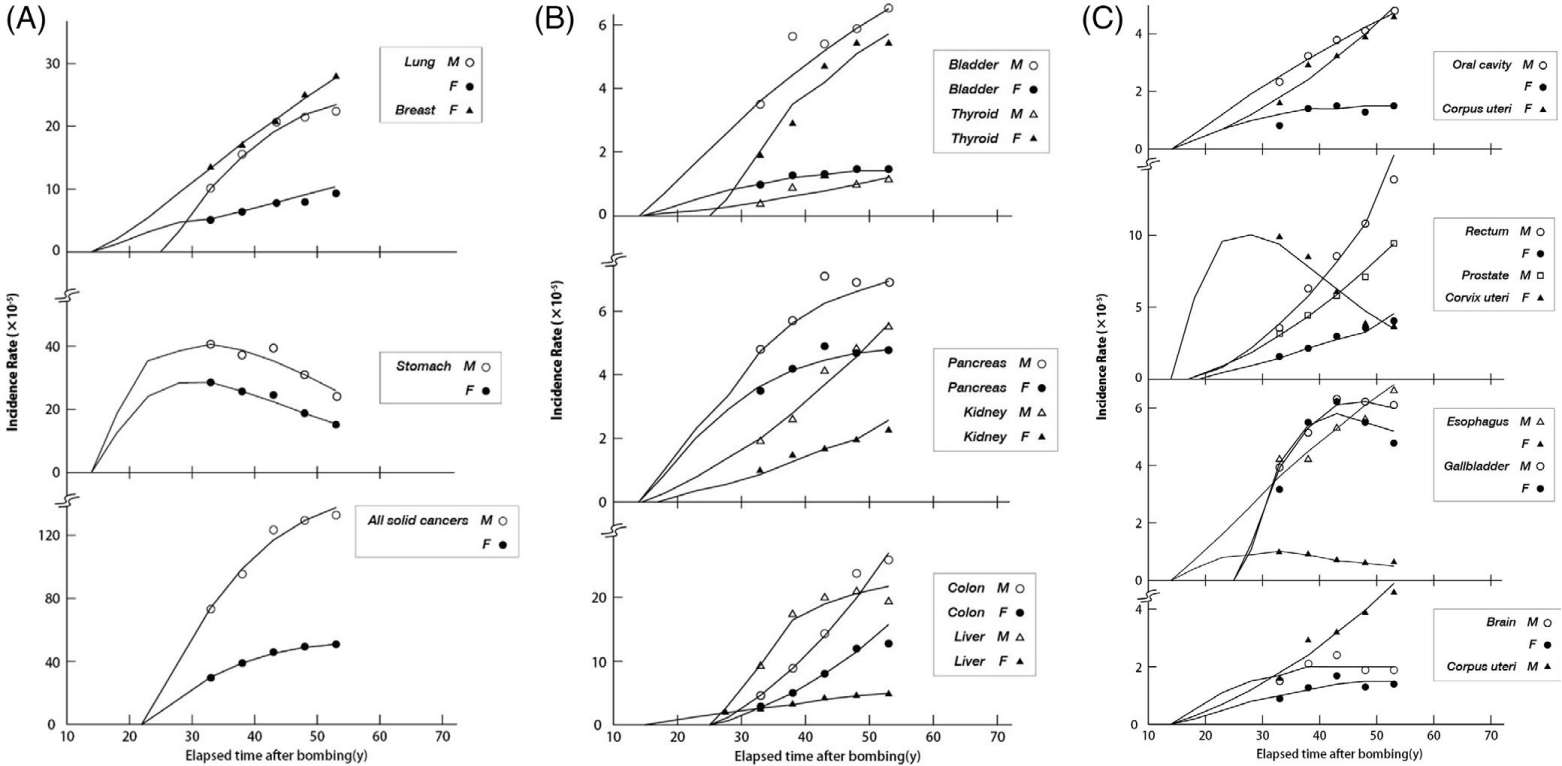
Observed and calculated incidence rates of net radiation‐induced solid cancers during 5 years for Hiroshima ‐Nagasaki atomic‐bomb survivors

After a steady and persistent effort, sufficient reproduction was achieved as shown in Figure 3A–C and deduced values of the parameters were summarized in Table 6. You may realize that the values of three main parameters, μ, Λ, and *B*, do not change before and after the subtraction of contribution of spontaneous cancers. This implies the skeleton of the present model is firm. Affected parameters are limited to the pathologically sensitive ones. This procedure does not reject the possibility of existence of equally likely solutions. Another set of values may be more probable for free parameters. However, our purpose is to demonstrate the existence of solution reproducing the observed incidence rates with reasonable fitness. Determination of the best set of values of parameters is not a primarily important matter. Even if you happen to find a different solution, it still stays within the framework of the present model. The resulting *Q*_sc_ values given in Tables 4 and 5 are quite reasonable considering the expected lower spontaneous incidence rate in Hiroshima−Nagasaki area compared with national incidence rate.^20^ This consequence would further reinforce model's justice.

**TABLE 6:** Parameters extracted for net radiation–induced incidences

Solid cancer		μ,Λ,β	*B*	ε	$f\left(\times 10^{-5}\right)$	$\mathrm{\gamma D}\left(\times 10^{-5}\right)$	$\alpha \left(\times 10^{-5}\right)$	Latent period (*y*)
Oral cavity	M	$\left(\begin{array}{c} \mu =0.05\\ \Lambda =0.01\\ \beta ≓\mu \end{array}\right)$	0.1	0.019	0.23	0.25	0.011	14
	F			0.042	0.22	0.24	0.011	
Esophagus	M			0.019	0.33	0.36	0.016	
	F			0.085	0.72	0.79	0.036	
Stomach	M			0.073	22.2	24.4	1.11	
	F			0.082	21.0	23.0	1.05	
Lung	F			0.035	0.97	1.1	0.049	
Pancreas	M			0.032	0.66	0.72	0.033	
	F			0.036	0.58	0.64	0.029	
Kidney	M			0	0.10	0.11	0.005	
	F			0	0.046	0.051	0.002	
Breast	F			0.015	1.1	1.2	0.055	
Cervix uteri	F			0.10	12.4	13.6	0.62	
Bladder	M			0.021	0.35	0.39	0.018	
	F			0.035	0.16	0.18	0.008	
Thyroid	M			0	0.021	0.023	0.001	
Brain	M			0.020	0.45	0.50	0.023	
	F			0.030	0.13	0.14	0.007	
Liver	F			0.020	0.26	0.29	0.013	
Corpus uteri	F			0	0.087	0.096	0.004	
Rectum	M		−0.3	0	0.10	0.07	0.005	17
Prostate	M			0	0.21	0.15	0.011	
Rectum	F		−0.5	0	0.35	0.18	0.018	19
Ovary	F			0	0.11	0.06	0.006	
All solid cancers	M		−0.7	0.041	33.5	10.1	1.68	22
	F			0.045	16.1	4.8	0.81	
Lung	M		−0.9	0.045	9.0	0.9	0.45	25
Liver	M			0.043	8.1	0.8	0.41	
Colon	M			0	1.02	0.06	0.051	
	F			0	0.58	0.7	0.029	
Gallbladder	M			0.066	7.3	0.7	0.37	
	F			0.085	11.2	1.1	0.65	
Thyroid	F			0.035	1.30	0.13	0.065	

## 5 MECHANISM OF SPONTANEOUS CANCERS IN THE DAILY LIFE

Now that the formulation of radiation−induced solid cancers is cleared, our next target is formulation of the daily−life spontaneous cancers. It is more serious problem to formulate the mechanism of spontaneous cancers, contrary to the former induced only in the emergency. However, any systematic yearly change in the incidence is not expected to appear among patients of spontaneous cancer contrary to the radiation−induced cancers. So, we must look for other sources of data on which we construct a mathematical model.

The risk of a cancer starts at the moment of birth and people keep staying under the risk thereafter. The age therefore corresponds to the exposed duration of Hiroshima−Nagasaki survivors. The data of yearly cancer incidence rates of age groups for 5 years given by National cancer center, Japan^20^ are our subjects to try to construct the mathematical model of the spontaneous cancer.

The devised model is based on the abnormal cell division. We adopted the yearly incidence rates^20^ of age groups for investigating the mechanism of spontaneous solid cancers. Age—dependent features of incidence rates reveal quite different aspects from the time dependence of radiation−induced cancers. The incidence rate stays at very low level below middle age and begin to increase quite violently thereafter.^18^ This lets us think of increase of the number of cells in the cell division and increase of abnormal cells produced at division and led to cancers.

However, the increasing rate of the number of cells is turned out too large to reproduce the incidence rate when we compute the number of cells with the condition that two cells are produced at each division. In order to avoid the catastrophe, we introduced programmed death of cell (apoptosis)^21^ to our model, as

(13) $$R\left(n\right)={\varsigma \eta }^{\kappa -n},$$

where *ζ* is the probability for occurrence of abnormal division at cell division, η is the number of surviving cells at cell division, and κ is the number of times of division occurring during a year.

This simple equation reproduces well the available incidence rates of 33 solid cancers and 4 data of Leukemia and Lymphoma given in reference 20 for the age groups from 20 to 50. However, calculated values still substantially exceed observed values for age groups above the 50. The possible way of avoiding this discrepancy may be attributed to the aging effect, which was introduced into Equation (13).

The discrepancy in Equation (13) should be attributed to either *κ* or η. As a result of examination, we reached a conclusion that only reduction of η for high generation can cover the discrepancy.

**FIGURE 4:**
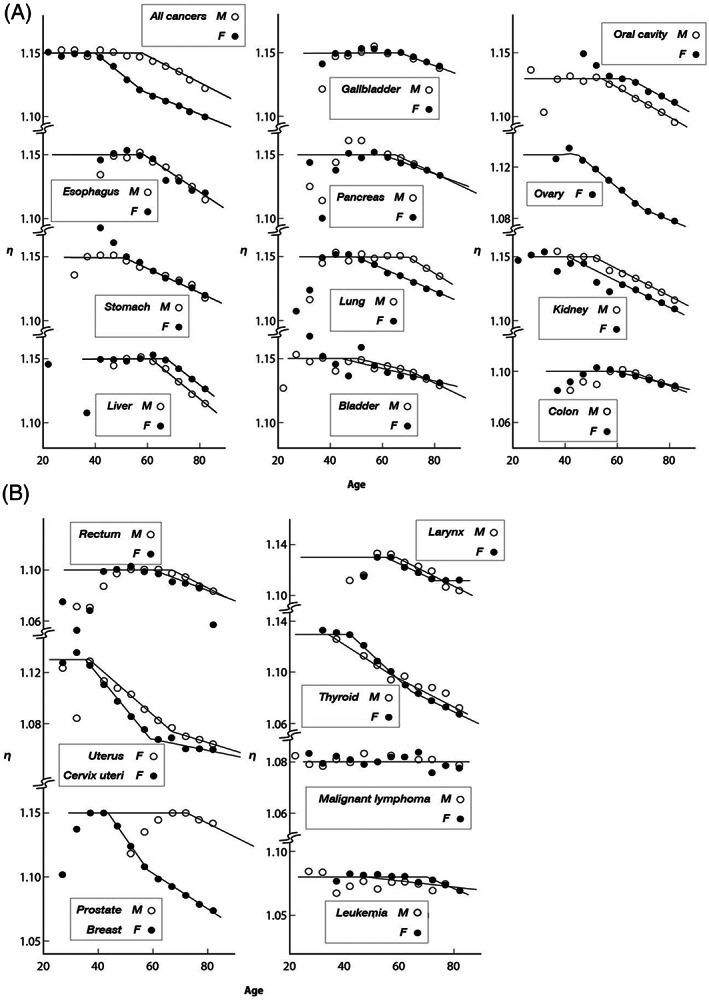
Age–dependence of the number of cells survived at cell division Equation (14)

It is then assumed that η stays constant until it starts to linearly decrease at the age *n*_0_ in the aging effect as shown in Figure 4A,B, namely

(14) $$\eta =\eta_{0}-\delta \;\phi \left(n-n_{0}\right),$$

for $n\leq n_{1}$_,_ where ϕ is a function defined as

(15) $$\phi \left(x\right)=\left\{\begin{array}{cc} 0 & \text{for}\;x\;≦\;0\\ x & \text{for}\;x\;>\;0 \end{array}\right.$$

and

(16) $$\eta =\eta_{1}-\delta_{1}\left(n-n_{1}\right)$$

for *n* > *n*_1_. The values of *n*_0_, *n*_1,_ *η*_0_, and $\eta_{1}$ are given in Table 7.

**TABLE 7:** Deduced parameters from incidence data of spontaneous cancers

		κ	$\eta_{0}$	*n*_1_	*n*_0_	*n*_1_	$\delta_{0}\left(\times 10^{-4}\right)$	$\delta_{1}\left(\times 10^{-4}\right)$	$\zeta \left(\times 10^{-5}\right)$
All solid cancers	M	0.9	1.150	–	58	–	11	–	0.63
	F			1.122	40	57	16	8.6	1.20
Esophagus	M			–	59	–	14	–	0.025
	F			–	59	–	14	–	0.0029
Stomach	M			–	51	–	9.7	–	0.19
	F			–	51	–	9.7	–	0.073
Liver	M			–	61	–	17	–	0.059
	F			–	67	–	16	–	0.013
Gallbladder	M			–	67	–	7.7	–	0.011
	F			–	67	–	7.7	–	0.0086
Pancreas	M			–	65	–	9.7	–	0.014
	F			–	61	–	7.7	–	0.0083
Lung	M			–	70	–	13	–	0.059
	F			–	51	–	6.9	–	0.034
Breast	F			1.103	43	61	9.3	14	0.43
Prostate	M			–	72	–	10	–	0.020
Kidney	M			–	51	–	11	–	0.021
	F			–	41	–	10	–	0.013
Bladder	M			1.140	50	71	4.8	10	0.019
	F			–	45	–	5.1	–	0.0046
Oral cavity	M		1.130	–	54	–	11	–	0.044
	F			–	65	–	11	–	0.0072
Larynx	M			–	59	–	11	–	0.020
	F			1.112	55	73	4.4	0	0.00097
Thyroid	M			–	35	–	13	–	0.040
	F			1.084	42	65	23	10	0.16
Uterus	F			1.074	36	62	19	6.3	0.40
Cervix uteri	F			1.068	35	59	26	4.5	0.34
Ovary	F			1.088	45	70	13	5.9	0.082
Colon	M		1.100	–	65	–	6.2	–	0.62
	F			–	61	–	4.7	–	0.38
Rectum	M			–	67	–	11	–	0.41
	F			–	59	–	8.0	–	0.22
Malignant lymphoma	M		1.080	–	–	–	0	–	0.26
	F			–	–	–	0	–	0.17
Leukimea	M			–	48	–	2.5	–	0.29
	F			–	70	–	9.3	–	0.15

The recursion work with age—dependent incidence rates averaged over 5 years from 1995 to 1999^20^ was repeated until getting a self−consistent solution for the introduced parameters for an appropriately given κ value. The best result was obtained when we chose the condition κ = 0.9 (13 months as the time between the adjacent divisions), respectively, and the value of *η*_0_ and the resulting functional form of η for each cancer is shown as a combination of a constant part and linearly decreasing section above a critical age *n*_0_ as given in Figure 4A,B.

**FIGURE 5:**
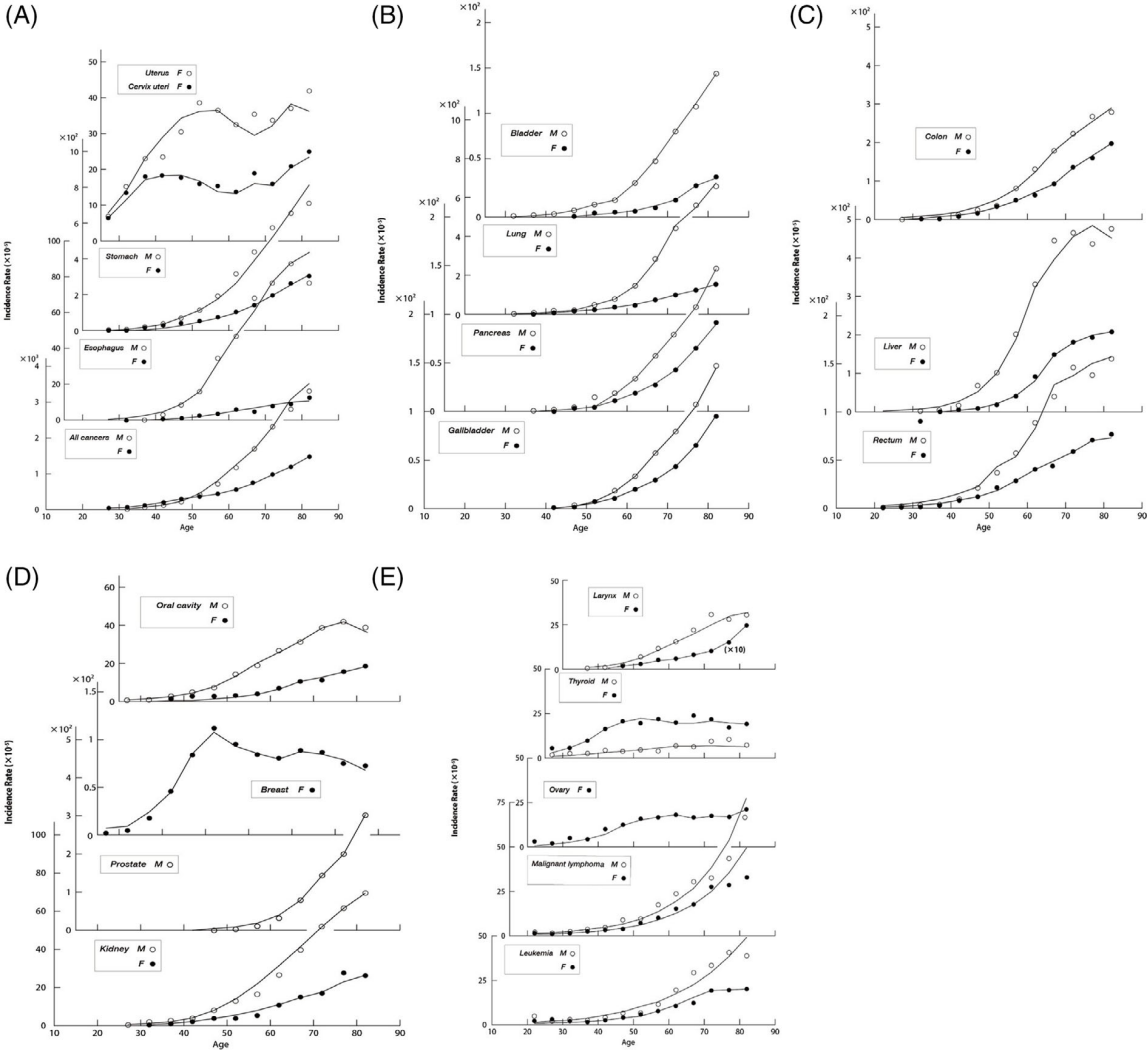
Observed and calculated incidence rates of whole spontaneous cancers

The resulting incidence rates are shown in Figure 5A–E in comparison with the observed values, and numerical data are given in Table 8. The deduced values for the introduced parameters are summarized in Table 7. All cancers were found controlled with two principal parameters, *κ* and *η*_0_. The *κ* was kept constant throughout all cancers. They were classified into four groups assigned with specific values of *η*_0_. Difference in the situation, such as site of cancer, sex, habits, medical treatment, and so forth, appears only in *ζ* and aging effects *η*, and have no influence on the fundamental structure of the mechanism of spontaneous cancers. It is notable that all cancers including breast, ovary, and uterus revealing irregular feature can be explained by a single equation. Furthermore, this model is valid not only for the solid cancer but also for general cancers.

**TABLE 8:** Observed and calculated incidence rate (number of incidence per 105 populations) for spontaneous cancers

Age (*y*)			20–24	25–29	30–34	35–39	40–44	45–49	50–54	55–59	60–64	65–69	70–74	75–79	80–84
All cancers	M	obs	17.7	20.1	37.0	62.4	127.6	237.8	408.7	708.1	1170.0	1765.2	2377.7	2806.5	3317.0
		cal	10.0	18.8	35.3	66.2	124.1	232.7	436.5	818.7	1264.3	1701.0	2307.1	3193.7	3514.7
	F	obs	19.5	34.4	67.4	126.5	216.1	306.3	367.0	449.9	571.7	766.4	999.0	1219.3	1497.6
		cal	19.1	35.8	67.2	126.0	214.1	295.2	381.3	440.4	576.2	763.6	978.9	1213.9	1455.7
Oral cavity	M	obs	0	1.0	1.0	2.5	4.7	7.1	14.2	19.0	26.9	31.1	38.6	41.5	38.9
		cal	0.5	0.9	1.5	2.6	4.5	7.7	13.4	20.3	25.8	31.2	38.1	41.8	38.1
	F	obs	0.4	1.1	0.2	1.2	2.7	2.7	3.5	4.1	6.7	10.5	11.2	15.1	18.4
		cal	0.1	0.1	0.2	0.4	0.7	1.3	2.2	3.8	6.6	10.3	12.5	15.4	18.2
Esophagus	M	obs	0	0	0	0.2	2.9	8.8	16.1	34.8	47.0	68.2	76.6	87.2	78.3
		cal	0.4	0.7	1.4	2.6	4.9	9.2	17.3	32.5	47.8	60.7	77.1	87.7	94.0
	F	obs	0	0	0	0	0.5	1.1	2.4	3.7	6.0	4.5	7.9	8.9	12.6
		cal	0.1	0.1	0.2	0.3	0.6	1.1	2.0	3.8	5.5	7.0	8.9	10.2	10.9
Stomach	M	obs	0.8	2.6	7.4	19.9	38.4	72.8	117.1	196.7	319.3	443.4	579.7	659.7	715.1
		cal	3.0	5.7	10.6	20.0	37.4	70.2	116.5	180.5	270.8	393.6	522.7	666.2	814.8
	F	obs	1.0	3.0	8.3	17.8	30.3	40.9	51.0	78.4	106.5	144.1	199.5	264.8	308.6
		cal	1.2	2.2	4.1	7.7	14.4	27.0	50.6	75.9	104.1	143.4	200.8	256.0	313.0
Colon	M	obs	1.2	1.4	2.8	4.7	13.6	26.0	35.6	83.8	133.4	181.4	225.3	271.0	283.1
		cal	4.1	6.3	9.6	14.8	22.8	35.9	53.6	82.4	126.5	183.9	222.0	259.2	292.5
	F	obs	0.2	0.3	2.7	5.7	10.7	19.7	37.3	52.7	66.8	96.5	138.5	163.5	201.5
		cal	2.5	3.9	5.9	9.1	13.9	21.4	32.9	50.5	73.7	95.6	136.1	169.3	205.3
Rectum	M	obs	0.2	0.8	3.0	4.1	9.9	21.5	37.6	57.5	89.4	116.4	146.4	138.7	155.6
		cal	2.7	4.2	6.4	9.8	15.0	23.1	44.1	54.5	83.7	128.5	138.4	150.9	157.7
	F	obs	0.2	1.3	1.0	2.8	8.0	12.7	21.8	28.8	40.2	44.4	59.6	71.5	77.6
		cal	1.5	2.2	3.4	5.3	8.1	12.4	19.0	29.2	40.6	49.6	58.6	71.3	73.9
Liver	M	obs	0.4	0.8	0.8	1.4	7.3	17.9	41.0	81.5	133.4	178.8	187.1	176.0	191.8
		cal	0.9	1.8	3.3	0.2	11.6	21.7	40.8	76.7	130.5	159.3	182.8	194.5	181.9
	F	obs	0.2	0	0.2	0.4	2.6	4.7	8.6	17.3	37.4	59.1	73.8	78.6	84.3
		cal	0.9	1.8	3.3	6.2	11.6	21.7	40.8	17.3	32.4	60.8	72.5	81.0	84.6
Gallbladder	M	obs	0	0.2	0.4	0.5	1.9	3.8	7.6	19.0	33.6	56.4	79.5	107.7	147.1
		cal	0.2	0.3	0.6	1.1	2.1	4.0	7.4	17.7	33.1	56.0	78.5	105.7	145.6
	F	obs	0	0.2	0.4	0.5	1.7	3.1	7.1	11.6	19.0	27.7	43.4	65.0	91.6
		cal	0.1	0.3	0.5	1.0	1.7	3.2	6.0	10.8	19.3	29.2	42.8	64.8	89.0
Pancreas	M	obs	0	0	0.4	0.5	2.2	7.4	14.6	19.0	33.6	56.4	79.5	107.7	147.1
		cal	0.2	0.4	0.9	0.9	11.6	3.1	5.8	17.7	33.1	56.0	78.5	105.7	145.6
	F	obs	0	0	0.4	0.2	1.1	3.2	5.3	11.6	19.0	27.7	43.4	65.0	91.6
		cal	0.1	0.2	0.5	0.9	1.6	3.1	5.8	10.8	19.3	29.2	42.8	64.5	89.0
Lung	M	obs	0	0.2	1.4	5.3	12.7	19.3	44.8	72.0	144.4	284.9	440.4	560.4	655.0
		cal	0.9	1.8	3.3	6.2	11.6	21.8	40.9	76.7	143.8	269.7	451.9	550.5	675.4
	F	obs	0	0.4	1.0	3.5	7.1	13.4	21.7	33.3	44.2	69.2	93.4	121.6	151.6
		cal	0.5	1.0	1.9	3.6	6.7	12.6	21.8	34.0	51.2	70.8	99.6	127.5	156.6
Breast	F	obs	1.4	4.5	17.3	45.7	83.4	111.5	95.2	83.4	80.1	88.5	86.6	74.7	72.7
		cal	6.8	8.2	24.1	45.2	84.7	109.8	94.0	82.9	79.3	86.8	85.0	78.3	67.9
Uterus	F	obs	1.1	6.8	15.2	23.0	23.5	30.5	38.5	36.3	32.4	35.3	33.7	37.0	41.9
		cal	4.5	7.8	13.5	22.7	28.0	33.1	34.6	34.9	30.9	35.0	36.2	35.8	36.3
Ovary	F	obs	3.5	2.6	4.8	4.4	10.0	12.4	16.0	17.5	18.7	16.1	17.4	19.8	20.3
		cal	0.9	1.6	2.8	4.8	8.3	12.4	15.8	17.3	18.5	16.5	17.2	17.0	20.9
Prostate	M	obs	0	0	0	0.1	0.1	0.1	3.1	11.2	32.5	77.7	143.3	197.8	303.7
		cal	0.3	0.5	1.0	1.8	3.3	6.3	11.8	22.1	41.4	77.7	145.8	202.1	306.3
Cervix uteri	F	obs	0.9	6.3	13.5	17.9	18.3	17.7	16.0	15.6	13.7	18.7	15.7	20.8	24.8
		cal	3.8	6.6	11.5	17.2	18.2	18.4	16.9	13.9	13.4	16.0	18.9	20.6	23.4
Bladder	M	obs	0.2	0.6	1.0	2.0	2.7	6.5	12.8	17.5	34.3	56.9	87.7	112.7	146.2
		cal	0.3	0.6	1.1	2.0	3.7	7.0	12.6	17.3	34.6	57.0	87.4	115.7	147.1
	F	obs	0.2	0	0.4	0.5	0.8	1.0	4.6	4.6	6.6	10.1	17.5	30.6	41.7
		cal	0.0	0.1	0.3	0.5	0.9	1.6	2.7	4.6	7.2	11.8	17.8	29.8	40.6
Kidney	M	obs	0.6	0.2	2.0	2.5	4.0	7.9	13.9	16.5	26.6	39.8	51.9	61.7	69.3
		cal	0.3	0.8	1.2	2.2	4.1	7.8	14.0	21.8	31.4	41.2	51.5	61.2	69.2
	F	obs	0.2	0.4	0.8	1.0	2.2	4.0	4.0	5.1	10.8	15.0	16.8	27.7	26.1
		cal	0.2	0.4	0.7	1.4	2.5	4.0	5.7	8.2	11.3	15.0	17.9	23.1	26.9
Larynx	M	obs	0	0	0	0.2	1.1	2.1	6.9	11.8	15.4	22.1	30.2	22.9	29.4
		cal	–	–	–	1.2	2.0	3.5	6.1	10.6	15.0	19.6	24.5	29.4	31.7
	F	obs	0	0	0	0	0	0.1	0.3	0.5	0.6	0.8	1.0	1.5	2.4
		cal	–	–	–	–	–	0.1	0.3	0.5	0.6	0.8	1.0	1.5	2.5
Thyroid	M	obs	0.6	1.7	2.4	2.3	4.1	3.7	4.4	4.0	7.0	6.5	9.3	10.3	7.1
		cal	0.4	0.8	1.4	2.1	2.9	3.7	4.7	5.6	6.7	6.8	7.0	6.8	6.3
	F	obs	4.4	5.5	5.9	9.6	16.1	20.4	19.5	21.7	19.6	23.8	21.8	17.1	19.2
		cal	1.8	3.1	5.4	9.4	16.2	20.1	22.1	21.3	19.6	19.6	20.8	19.8	19.2
Leukimea	M	obs	5.0	2.1	2.9	2.5	4.0	6.5	7.0	11.6	19.6	29.4	33.6	40.7	39.0
		cal	1.3	2.1	2.7	3.8	5.3	7.5	10.6	13.0	17.3	22.7	29.6	38.3	49.1
	F	obs	1.9	3.0	2.5	1.7	2.9	4.1	6.1	7.8	10.9	12.4	19.3	19.8	20.3
		cal	0.7	1.0	1.4	1.9	2.8	3.9	5.0	7.8	11.0	15.5	19.5	19.8	20.6
Malignant lymphoma	M	obs	1.3	1.6	2.2	3.6	4.8	8.8	9.6	17.3	23.8	30.1	42.7	43.7	66.6
		cal	1.2	1.7	2.4	3.4	4.8	6.7	9.5	13.5	19.1	26.9	38.1	53.9	76.1
	F	obs	1.5	1.3	1.5	2.5	3.3	4.0	7.3	10.2	15.2	17.9	27.6	28.8	38.1
		cal	0.8	1.1	1.6	2.2	3.1	4.4	6.2	8.8	12.5	17.6	24.9	35.2	49.8

## 6 DISCUSSION

The present model for radiation−induced cancers satisfactorily reproduces the incidence rates of all reported cancers without any exception and therefore would have strong persuasiveness. According to this model, observed strong immune effects are explained by means of destruction of immune−restraint cells by rather low−level environmental radiation during several 10 years after the bombing, not by radiation of high dose at the moment of bombing. This implies that immune−restraint cells possess only very low tolerance against radiation.

The most noteworthy item in Table 6 is the result that μ and Λ take common values, respectively, throughout the cancers. The fact that their values are the same for all cancers indicates the processes controlled by *μ* and Λ are the purely mechanical process nevertheless their pathological differences. On the other hand, the probability *f* of producing radiation damage and the parameter ε giving the immune effect respectively take a different value for individual cancer and concluded to be controlled by biological or pathological factors.

The parameter ε is considered to quantitatively reveal the existence of hormesis effect, which has been favorably discussed for a long time among health physicists by experience and suggests the possibility of preventive treatment of cancers. Verification of the hormesis effect may be carried out rather easily within a short time.

The model constructed for spontaneous cancers explains well the observed incidence rates. Furthermore, the physical scenario does not contradict the two−stage clonal expansion^2^ or multi−stage carcinogenesis model^22^ for cancers. Formulation of spontaneous cancers leads to the conclusion that the cancer is induced with the cells lost the function of apoptosis at abnormal cell division. In general, apoptosis is explained as a phenomenon occurring on the useless or incomplete cells. Apoptosis called in the present model requires the function that cells generated at the cell division are always extinguished with the definite and unexpectedly large ratio.

Now we can get the answer to the question what the prodromal cell is received damage by radiation. The damage given by radiation is so trivial compared to the damage produced in the segmentation that it is unable to deprive the function of apoptosis. Mutation from the unstable prodromal cell takes place during the latent period, which is generally observed among radiation‐induced cancers. These observations unify the two apparently independent models to one framework. The character of mutation in the second stage of radiation−induced cancer corresponds to the abnormal cell division of spontaneous cancers and the parameter *β* corresponds to *ζ* of spontaneous cancer. The former is larger than the latter by two to three figures. This indicates high enhancement of cancer incidence by low−level radiation.

This model proceeds to an idea that apoptosis is the very essential function for the proper growth of creature and continual existence of creature itself is threatened if this function has ceased to work. The part of apoptosis occupying in the evolution of creation is then realized to be far more important than being imagined so far.

The expected hormesis effect suggests that the possible preventive treatment of low−dose irradiation before generation of cancers may be effective for at least half of the observed 37 cancers possessing large ε values given in Table 6. Moreover, the hormesis effect is considered to be a promising measure of suppressing metastasis for all solid cancers.

Medical treatment of low−dose irradiation for whole body would be effective for killing damaged cells at pre‐cancer stage. Fortunately, the hormesis effect is particularly strong for cancers of stomach, lung, liver, and so forth, showing high incidence rates. Therefore, the incidence rate would be suppressed to a large extent, when this treatment is accepted in a nationwide scale.

When this treatment is proved effective, patients once suffered by cancer will be relieved from the fear of recurrence of cancer without any severe side effect. Recently, it is reported that there are females who cut off their sound breast to avoid the future hazard of breast cancer. Female adults will certainly be liberated from such a bitter judgment.

If the present model is correct, the proposed medical treatment with low−dose radiation is expected to be applicable to other cancers, for instance leukemia. Then, the patients would be relieved from severe surgical treatment like marrow transplantation. We hope that low−dose irradiation technic is developed and becomes widespread in future as a preventive treatment for cancers without ill effects.

## AUTHOR CONTRIBUTIONS

**Hiroshi Baba:** Conceptualization (lead); data curation (lead); formal analysis (lead); funding acquisition (lead); investigation (lead); methodology (lead); project administration (equal); software (supporting); supervision (equal); visualization (equal); writing – original draft (lead); writing – review and editing (equal). **Akihiko Yokoyama:** Conceptualization (supporting); data curation (supporting); formal analysis (supporting); funding acquisition (supporting); investigation (supporting); project administration (equal); software (lead); supervision (equal); visualization (equal); writing – original draft (supporting); writing – review and editing (equal).

## CONFLICT OF INTEREST

The authors have no conflict of interest to be disclosed related to this work.

## EHICS STATEMENT

This research proposes a mathematical model based on published data, and does not raise ethical issues.

## Data Availability

Data availability statement is not applicable for this study.
